# Modeling Stone Columns

**DOI:** 10.3390/ma10070782

**Published:** 2017-07-11

**Authors:** Jorge Castro

**Affiliations:** Department of Ground Engineering and Materials Science, University of Cantabria, Avda. de los Castros s/n, 39005 Santander, Spain; castrogj@unican.es; Tel.: +34-942-201813

**Keywords:** stone columns, encased stone columns, geosynthetic, numerical modeling, critical length, parameter selection

## Abstract

This paper reviews the main modeling techniques for stone columns, both ordinary stone columns and geosynthetic-encased stone columns. The paper tries to encompass the more recent advances and recommendations in the topic. Regarding the geometrical model, the main options are the “unit cell”, longitudinal gravel trenches in plane strain conditions, cylindrical rings of gravel in axial symmetry conditions, equivalent homogeneous soil with improved properties and three-dimensional models, either a full three-dimensional model or just a three-dimensional row or slice of columns. Some guidelines for obtaining these simplified geometrical models are provided and the particular case of groups of columns under footings is also analyzed. For the latter case, there is a column critical length that is around twice the footing width for non-encased columns in a homogeneous soft soil. In the literature, the column critical length is sometimes given as a function of the column length, which leads to some disparities in its value. Here it is shown that the column critical length mainly depends on the footing dimensions. Some other features related with column modeling are also briefly presented, such as the influence of column installation. Finally, some guidance and recommendations are provided on parameter selection for the study of stone columns.

## 1. Introduction

Ground improvement using stone columns, also known as granular piles or aggregate piers, is one of the most popular techniques to improve soft soils for the foundation of embankments or structures. These are vertical boreholes in the ground, filled upwards with gravel compacted by means of a vibrator.

The idea of improving soft soils for foundation purposes using granular inclusions is relatively old. It is documented [1] that in 1839 in Bayonne (France), the French colonel Burbach used for the first time sand piles as deep foundations instead of the classical wood piles that rapidly degrade with fluctuations of the ground water level. However, it was not until the 50 s of the last century when stone columns started to be used. The ground improvement technique started as an extension of traditional vibro-compaction (deep compaction) to non-granular soils, whose low permeability and cohesion do not allow for a quick rearranging of soil particles in a denser configuration.

Stone columns act mainly as inclusions with a higher stiffness, shear strength and permeability than the natural soil. Consequently, they improve the following aspects:

- The bearing capacity
- The stability of embankments and natural slopes
- Final settlement
- Degree of consolidation
- Liquefaction potential

The reduction of the liquefaction potential is beyond the scope of the present paper. Please refer to [2,3] for further information on that topic. The present paper focuses on the other four improvements, particularly, the settlement reduction. The modeling strategy for stone columns should be chosen depending on which of the previous improvements is to be analyzed.

In extremely soft soils, stone columns are not suitable because their continuity, stability, geometric shape, etc. cannot be guaranteed. The undrained shear strength ($c_{u}$) of natural soft soil is generally used as the limiting parameter for stone column feasibility. A limiting value around 5–15 kPa [4] may be adopted. To improve the lateral confinement of stone columns in those extremely soft soils, encasing the columns with geotextiles or other geosynthetics, such as geogrids, has proven to be a successful technique in recent times (e.g., [5]). Rigid or semi-rigid inclusions (e.g., adding lime or cement) is another common alternative (e.g., [6,7]). Han [8] summarizes different ground column technologies.

Ground improvement using stone columns, either ordinary or encased stone columns, requires a considerable amount of columns or, at least, a group of columns. This implies a complex modeling process of the real geometry. This paper provides some guidelines for obtaining these simplified geometrical models. Some other features related with column modeling are also briefly presented, such as the critical length of the column and the influence of column installation. Additionally, some guidance and recommendations are provided on parameter selection to study stone columns. Here, the word “modeling” is understood in a broad sense, covering geometrical, mechanical, geotechnical and installation features of stone columns.

The increase in computer power and the availability of finite element codes makes numerical analyses very appealing in geotechnical design. They usually lead to more detailed studies but require a clear conception of the modeling techniques. Ground improvement techniques, such as stone columns, are more and more popular due to the increasing occupation of natural soft soils and environmental concerns [9,10]. Within these current trends, the review of the modeling techniques for stone columns seems interesting and useful. Besides, in some instances, there is some confusion about geometrical models and their application. For example, the results for an isolated column under concentrated load just on top of the column cannot be directly extrapolated for a large group of columns under distributed uniform load.

## 2. Geometrical Models

**Figure 1:**
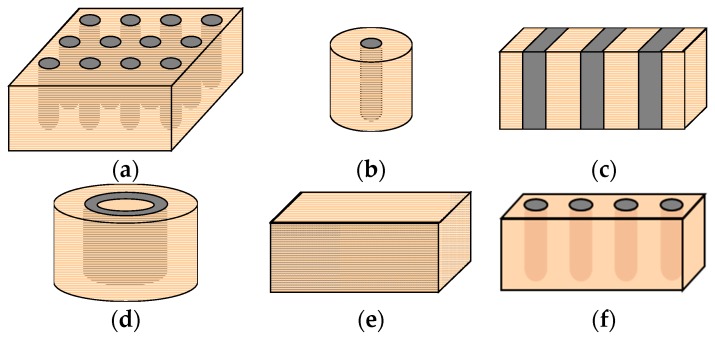
Main geometrical models for stone column studies: (**a**) Full 3D model; (**b**) Unit cell; (**c**) Longitudinal gravel trenches; (**d**) Cylindrical gravel rings; (**e**) Equivalent homogenous soil; (**f**) 3D slice of columns.

To simplify the real geometry of the problem that usually involves a considerable amount of columns (e.g., Figure 1a) and to be able to deal with the problem, the following simplified geometrical models are usually adopted:

- “Unit cell” in axial symmetry (Figure 1b). Only one column and its corresponding surrounding soil are studied. It may be useful to study just a horizontal slice of the unit cell, rather than the whole length.
- Longitudinal gravel trenches (Figure 1c). The stone columns are transformed into longitudinal gravel trenches to study the problem in plane strain conditions.
- Cylindrical rings of gravel (Figure 1d). The columns are transformed into cylindrical rings of gravel to study the problem in axial symmetry.
- Homogenization or equivalent homogeneous soil (Figure 1e). The columns and the surrounding soil are transformed into a homogeneous soil with equivalent improved properties.
- Three-dimensional (3D) model of a row or slice of columns (Figure 1f).
- Geometrical models for small groups of stone columns.
- “Unit cell” with constant lateral pressure (triaxial conditions).
- Isolated column.

The two latter cases, namely “unit cell” under triaxial conditions and isolated column, do not usually appear in real problems, but they have been used for laboratory tests (e.g., [11,12,13]). The case of an isolated column is widely used for field tests (usually plate load tests) for the sake of simplicity (e.g., [14]).

As a simple introductory comparison between the different geometrical models, Table 1 summarizes the suitability of some of these models to study the improvements achieved with a stone column treatment for the foundation of an embankment.

All the geometrical models listed above are valid for non-encased stone columns. However, for encased stone columns, there is not yet any satisfactory way to convert the cylindrical encasement (geosynthetic) that surrounds the column for the cases of longitudinal gravel trenches and cylindrical rings of gravel.

## 3. Unit Cell

The unit cell model is the most widely used for theoretical analyses and it is reviewed in detail, for example, in [15]. The basis for the simplified model is the usage of a great number of columns, uniformly distributed in a wide area under a uniform load. This is the case, for example, in the central part of an embankment on soft ground improved with stone columns. In these situations, the behavior of all the columns is the same and then, it is enough to study the behavior of just one column with the corresponding surrounding soil (tributary area). Due to symmetry conditions, at the external lateral boundary of the unit cell, only vertical displacements and only vertical water seepage are allowed.

**Figure 2:**
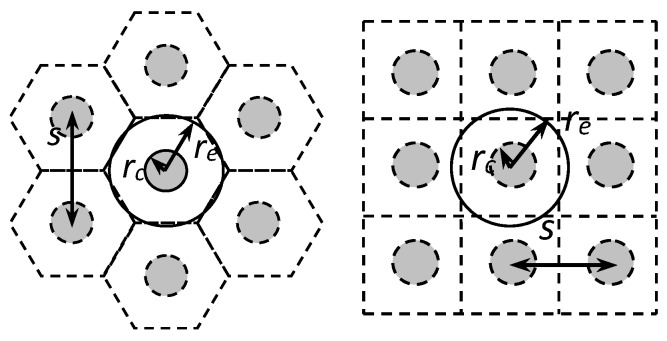
Simplification of the unit cell to axial symmetry conditions.

**Figure 3:**
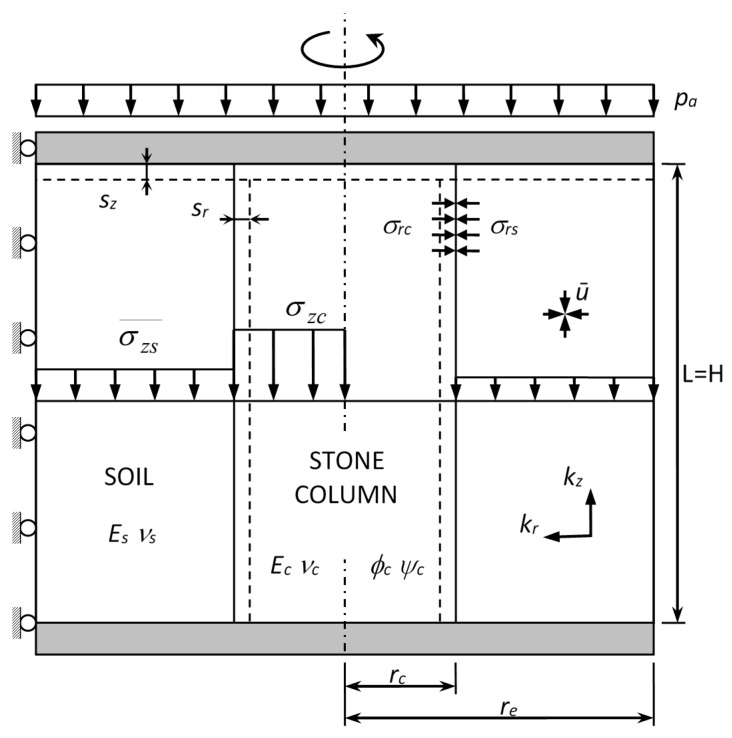
Unit cell model for analytical solutions.

Stone columns are generally uniformly distributed in triangular or square grids. Thus, the tributary area of natural soil for each column is a hexagon or a square, respectively. To allow for axial symmetry conditions, the tributary area is transformed into a circle (cylinder) of the same (cross-sectional) area. Therefore, the diameter of the unit cell is equal to $d_{e}=1.05-1.13\;s$ for triangular and square grids, respectively, where s is the centre-to-centre distance between columns (Figure 2). Figure 3 shows the unit cell model with axial symmetry that can be studied in two dimensions.

The unit cell model is used for most of the existing analytical solutions (e.g., [16,17]). In the analytical solutions, there is usually a further simplifying assumption for the geometry, which is to assume that the behavior of each horizontal slice is independent, i.e., the shear stresses are neglected. Numerical analyses [17,18] show that shear stresses are usually negligible for distributed loads. Balaam and Booker [19] is a notable exception to the simplifying assumption of neglecting shear stresses because they study the total length of the unit cell as a whole. Nevertheless, the solution requires numerical integration, which makes the solution complex for practical purposes.

Most analytical solutions focus on the settlement reduction caused by stone columns (e.g., [16,17,19]), but stone columns also act as vertical drains and, therefore, they dissipate excess pore pressures. The consolidation process may be studied independently from the settlement analysis using solutions for vertical drains [20,21]. Han and Ye [22] and Castro and Sagaseta [23] showed that the consolidation process around stone columns is slightly different because of the distribution of vertical stresses between soft soil and stone columns and they proposed specific analytical solutions to study the consolidation process around stone columns. Very recently, Pulko and Logar [24] have developed a fully coupled solution for the consolidation process assuming the soil as a poroelastic medium and the stone column as an elastoplastic material. The solution is very accurate but requires numerical inversion of the Laplace transform.

**Figure 4:**
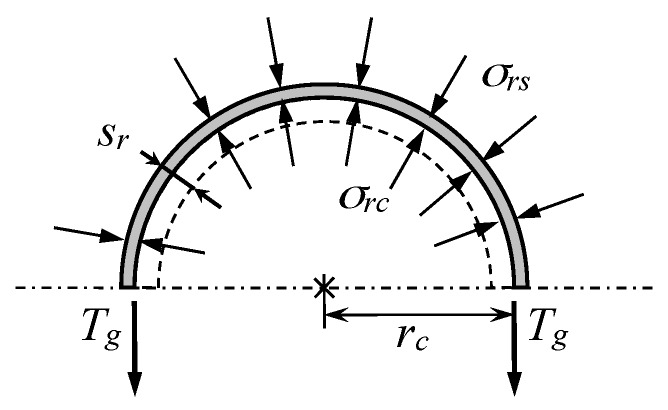
Equilibrium and compatibility conditions for the geosynthetic surrounding an encased stone column.

Extending analytical solutions for ordinary stone columns to encased stone columns is quite straightforward (e.g., [25]). Equilibrium and compatibility conditions of the geosynthetic encasement are those of a thin-walled tube, or better said, those of a flexible membrane because the encasement does not usually support compressive stresses. The internal and external pressures are here denoted as $\sigma_{rc}$ and $\sigma_{rs}$, respectively (Figure 4). Thus, the equilibrium and compatibility conditions of the geosynthetic encasement are the following, respectively:

(1) $$\sigma_{rc}=\frac{T_{g}}{r_{c}}+\sigma_{rs}$$

(2) $$T_{g}=J_{g}\frac{s_{r}}{r_{c}}$$

where $s_{r}$ is the radial displacement of the column and the encasement, $J_{g}$ is the hoop or circumferential stiffness of the geosynthetic and $T_{g}$ is the hoop force at the geosynthetic. The units of $J_{g}$ and $T_{g}$ are force per length (*F*/*L*) because the thickness of the geosynthetic is usually negligible. The equation that relates the radial stress of the column ($\sigma_{rc}$) with that of the soft soil ($\sigma_{rs}$) and allows the confining effect of the encasement to be included is obtained by combining Equations (1) and (2):

(3) $$\sigma_{rc}=\frac{J_{g}s_{r}}{r_{c}^{2}}+\sigma_{rs}$$

The unit cell model is also very useful for numerical analyses because numerical simulations allow more complex features to be studied than with the analytical solutions, such as layered ground, advance constitutive models or cyclic loads (e.g., [26,27,28,29]). Nowadays, 3D numerical codes are fairly easy to access and to use; so, there are some authors that study the unit cell as a full 3D problem, i.e., considering the hexagonal or square prism. Nevertheless, the differences between the full 3D prism and the two-dimensional (2D) model (cylinder) in axial symmetry are negligible [30,31]. The simplicity of the unit cell model has recently led to highly advanced numerical models, such as that presented by Indraratna et al. [32], where the column is modelled using 2D discrete elements to represent the gravel particles. This type of numerical analysis can be regarded only as explorative and for research purposes. The unit cell model or the model of a slice of the unit cell at a specific depth are also used for small-scale laboratory studies (e.g., [33,34]).

**Figure 5:**
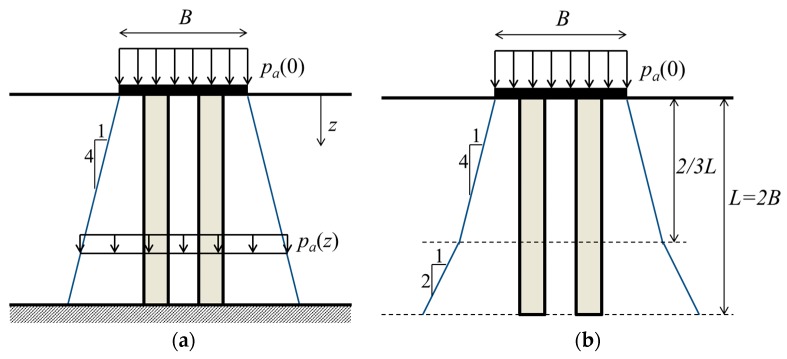
Stress distribution beneath footings: (**a**) End-bearing columns; (**b**) Floating columns.

The unit cell also allows floating columns to be studied, i.e., columns that do not reach a rigid substratum. In this case, the settlement due to column punching into the underlying layer and the deformation of the underlying layer should be considered [35]. The unit cell model is only valid when the ratio between the width of the loaded area and the thickness of the soft soil layer (B/H) is high enough to ensure confined (oedometric) conditions. On the contrary, the applied load is distributed with depth, in a roughly trapezoidal manner, its value decreasing with depth. Figure 5 presents some specific proposals of load distribution width depth for end-bearing and floating columns [36,37,38]. The unit cell model for these cases usually overestimates the settlement because the reduction of the applied vertical stress with depth is not reproduced.

Finally, the unit cell is an appropriate simplified geometrical model to study the settlement and its evolution with time at the center of an embankment, but obviously it is not valid for studying the stability of the lateral slopes (Table 1). For similar reasons, it does not allow the lateral spreading and the contribution of a geosynthetic reinforcement that acts as a “blanket” or “bridging layer” over the columns and soft soil foundation (geosynthetic reinforced and column supported embankments, GRCSE) to be studied [31]. To study the lateral spreading of the embankment, the unit cell model may be improved by substituting the horizontally-fixed external lateral boundary by elastic springs [39].

## 4. Longitudinal Gravel Trenches

**Figure 6:**
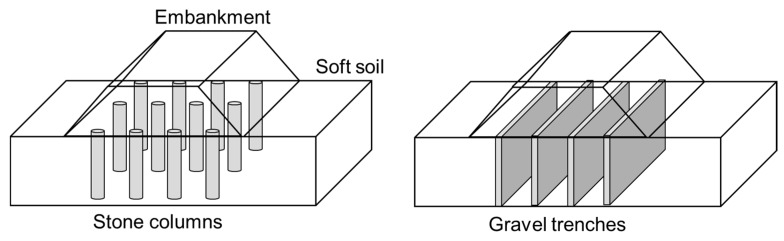
Longitudinal gravel trenches to model a stone column treatment in plane strain.

Many geotechnical problems fulfill plane strain conditions. Thus, when stone columns are used in those problems, for example, for the foundation of an embankment for a linear infrastructure, it is useful to study the problem under 2D plane strain conditions, transforming the columns into longitudinal gravel trenches (Figure 6). The width of the trenches and the spacing between them are part of the unknown parameters that should be properly estimated to be equivalent. Besides, it is usually necessary to alter the gravel parameters for these equivalent gravel trenches. Furthermore, to appropriately model the consolidation process, it is also necessary to modify the permeability of the natural soft soil.

### 4.1. Settlement

The classical proposal for this simplified model is by Van Impe and De Beer [40], who developed an analytical solution to study the settlement for this case. Their proposal [40] is to keep the (drained) properties of the soil and column and transform each row of columns in a longitudinal gravel trench of the same area.

The most important parameter in a stone column treatment is the area replacement factor ($a_{r}$), which represents the area of soft soil replaced or displaced by the stone columns:

(4) $$a_{r}=\frac{A_{c}}{A_{e}}=\frac{r_{c}^{2}}{r_{e}^{2}}$$

**Figure 7:**
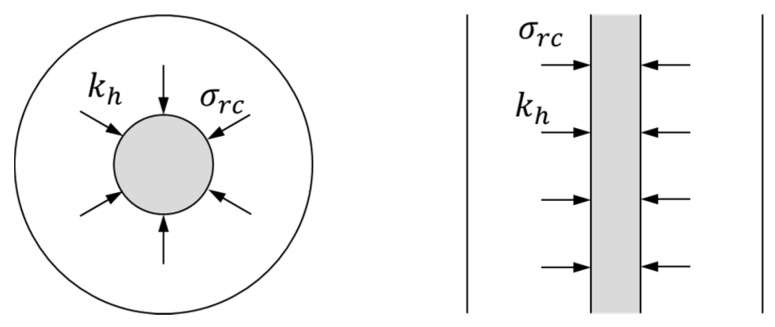
Different confinement and seepage conditions for columns in axial symmetry and for longitudinal gravel trenches in plane strain.

The proposal by [40] seems reasonable because it allows the area replacement factor ($a_{r}$) and the soil and gravel parameters to be kept. However, it has two major disadvantages:

- The resulting thickness of the gravel trenches is usually small, and consequently, the gravel trenches are very slender.
- The confining conditions of the columns are not the same as the lateral confinement of the gravel trenches [30] (Figure 7).

Regarding the first bullet point, there is no restriction on increasing the thickness of the gravel trenches, while proportionally increasing the spacing between the longitudinal trenches to keep $a_{r}$ constant. A reasonable value for the thickness of the gravel trenches may be the diameter of the columns.

**Figure 8:**
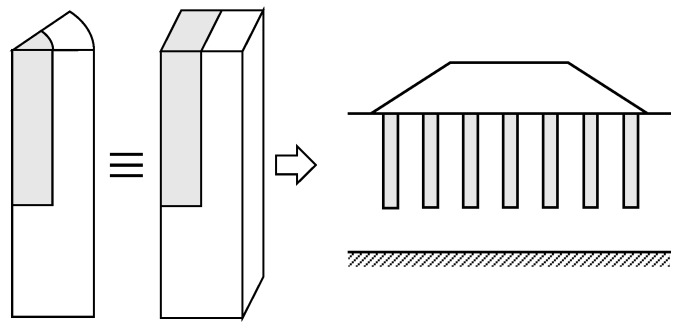
Calibration of the parameters of longitudinal gravel trenches using the unit cell model as an auxiliary problem.

The second bullet point causes that the matching of the settlement between the real situation and the plane strain model is not accurate enough, particularly when plastic strains appear in the columns and then, the differences in the column confinement are notable. Plastic strains in the columns are common and, if the column only deforms elastically, the design is overconservative. Tan et al. [30] presented two analytical proposals to obtain the equivalent properties of the gravel trenches and the surrounding soil, but neither of them is totally satisfactory. An analytical equation may be derived for the equivalent elastic modulus of the gravel trenches [41], but that is only valid if the column and the soil deform elastically. For general cases, comparing the unit cell model in axial symmetry and the unit cell of the gravel trenches is very useful (Figure 8). Thus, starting from an analytical proposal for the parameters of the gravel trenches, they may be further adjusted or tuned to match the settlement of the unit cell in axial symmetry. To fit the settlement in the plastic range, adjusting the friction angle of the gravel trenches seems the most appropriate alternative [41]. Once the parameters have been calibrated using the unit cell case as an auxiliary problem, they may be used for the full 2D plane strain model. However, the calibration is tailor-made or specific for each case. Thus, it varies, for example, with the value of the applied load [42].

### 4.2. Consolidation

The coefficient of horizontal permeability $k_{h}$ (or the coefficient of consolidation $c_{vh}$) of the natural soil should be adjusted to properly reproduce the consolidation process under plane strain conditions because, similarly to the column confinement, the seepage problem is different in axial symmetry and plane strain conditions (Figure 7). Hird et al. [43] and Indraratna and Redana [44] proposed analytical expressions to modify $k_{h}$, based on the comparison between analytical solutions for vertical drains for both cases (axial symmetry and plane strain).

When using numerical analyses, the analytical values of $k_{h}$ [43,44] may be tuned using the unit cell as an auxiliary problem (Figure 8) to have a better matching of the results. As it is difficult to match the whole consolidation curve, it may be convenient to match the values of the degrees of consolidation that are relevant for the study, usually around 80–95%.

### 4.3. Stability

The plane strain model is useful for studying the global stability, or, for example, the stability of the lateral slopes of an embankment (Table 1). In this case, the plane strain model should match the average shear strength along the slip line of the real situation. Here, it is important to distinguish between limit equilibrium analyses (e.g., method of slices) or full stress-strain analyses (e.g., finite differences or finite element method).

Stress-strain analyses are able to capture the stress concentration on the columns. Thus, if the stress concentration on the columns is properly reproduced (depending on the rigidity of soil and gravel), the results are satisfactory just keeping constant the area replacement ratio $a_{r}$ and the resistance properties (shear strength) of soil and gravel (e.g., [45]).

For limit equilibrium analyses, the stress concentration on the longitudinal gravel trenches should be artificially generated because the vertical stress is directly the weight above the studied point. Priebe [35] proposed altering the real profile of the embankment, so the modeled height of the embankment is the real one times the stress concentration either on the columns or on the soft soil. Another alternative is to modify the friction angle of the longitudinal gravel trenches to match the average shear strength along the slip line [15].

A very difficult task is to evaluate the stress concentration on the columns because it depends on many factors, such as the drainage conditions (undrained, partial drainage or fully drained), the depth and inclination of the slip line, the specific position of the columns beneath the slope and the applied load. Recent studies [45] show that in situations close to failure, there is no stress concentration on the columns, i.e., the vertical stress on soil and columns is very similar. Thus, assuming no stress concentration may be generally advisable and in any case, it is an assumption on the safe side.

Finally, rigid inclusions, such as deep mixing columns, may fail not only by shearing as stone columns (shear failure along the slip line), but they may also fail by tilting or bending [46]. For widely spaced columns, soil may also extrude between columns [47]. When columns break by bending, it is necessary to consider their moment of inertia [48] or the measured bending failure for encased stone columns.

## 5. Homogenization

The homogenization method consists in replacing the stone columns and the soft soil by an equivalent homogeneous soil with improved properties. This equivalent homogeneous soil occupies the zone treated with stone columns. This model simplifies enormously the geometry of the problem. For example, when the problem itself has a highly complex geometry and the zone treated with columns is just a part of the problem, this method is highly advisable (e.g., [49]). For the design stage, this method allows the area replacement ratio ($a_{r}$) to be varied without changing the geometry of the model, just the material parameters.

### 5.1. Equivalent Parameters

The most straightforward proposal for obtaining the improved parameters of the equivalent homogeneous soil is just to average the soil and column parameters weighted by their corresponding areas though $a_{r}$. Thus, for the elastic modulus, the weighted average is:

(5) $$E_{m}=E_{s}\left(1-a_{r}\right)+E_{c}a_{r}$$

However, this can only be regarded as a first approximation. A more detailed analysis shows that the equivalent homogenous soil should be anisotropic by nature to account for the column orientation [50]. Some authors (e.g., [51,52,53]) use theories for periodic media to propose analytical transformations from the columns and the soft soil (heterogeneous periodic composite material at the microscale) to a homogeneous equivalent soil (homogenous material at a macroscale). They use a macroscopic strength criterion of the anisotropic homogeneous equivalent soil to evaluate the bearing capacity. The practical application of these techniques is complex and they are only valid for sufficiently small values of the scale factor, i.e., the ratio between the micro and macroscales, for example, the ratio between the column spacing and the characteristic size of the whole problem to be studied.

**Figure 9:**
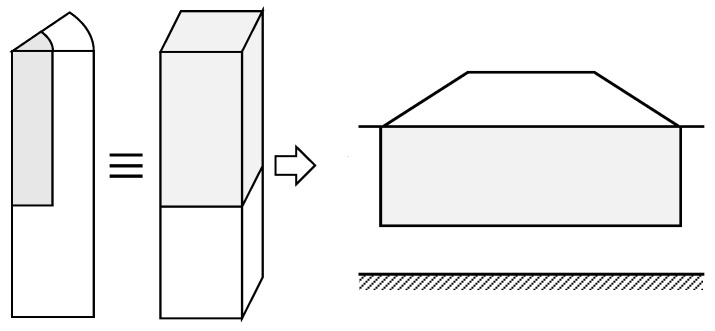
Calibration of the parameters of the equivalent homogeneous soil using the unit cell model as an auxiliary problem.

**Figure 10:**
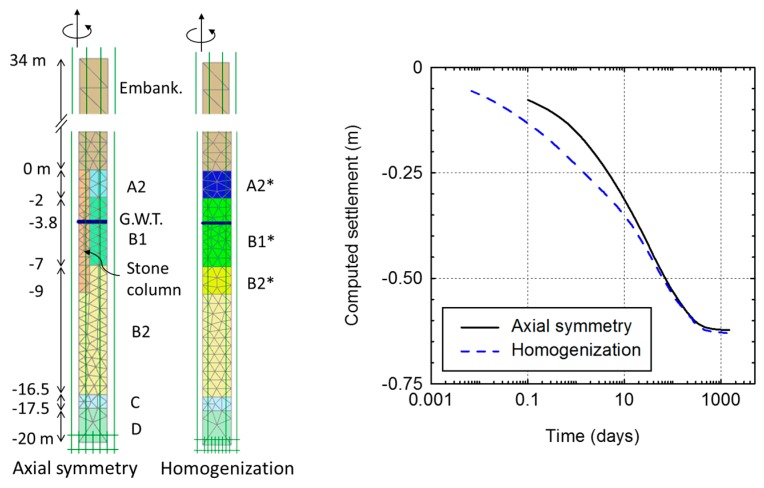
Example of calibration of the parameters of the equivalent homogeneous soil using the unit cell model as an auxiliary problem.

In a similar manner as for the longitudinal gravel trenches, a simple method when using numerical analyses is tuning or adjusting the improved parameters of the equivalent soil using the unit cell as an auxiliary problem (Figure 9). Ng and Tan [54] have calibrated the improved parameters for several cases and tabulated their results as a reference. The accuracy of the matching may be high and, yet the specific values of the excess pore pressure between columns may not be modeled using the homogenization technique, the average degree of consolidation or the settlement rate may be correctly capture after a proper calibration of the parameters of the equivalent homogeneous soil [54]. For most cases, it may be enough to match just the values of the degree of consolidation that are relevant for the study, usually around 80–95%. Figure 10 shows a simple example of application for the extension of an airport runway, whose embankment had to be founded on ground improved with floating stone columns.

The homogenization method cannot reproduce physical phenomena at the local level, such as stress concentration on the column or radial drainage towards the column, but the overall response of the system on a larger scale may be correctly reproduced after calibrating the parameters of the equivalent homogeneous soil.

### 5.2. Specific Constitutive Model

The idea of developing an advanced, specific constitutive model to represent the behavior of the periodic material formed by the columns and the corresponding surrounding soil was initially formulated by Schweiger and Pande [55]. The constitutive model assumed that both soil and column undergo the same strains, there is no slippage between soil and column and the model internally uses independent and existing constitutive models for the soil and the column and ensures that there is equilibrium of horizontal stresses at the soil-column interface [55]. The model was later improved, particularly its numerical implementation [56]. Recently, the idea has been applied to deep soil mixing columns using advanced constitutive models and with the aim of using it for complex 3D geometries [57]. However, the numerical stability of its formulation and its range of application are still limited.

## 6. Gravel Rings

**Figure 11:**
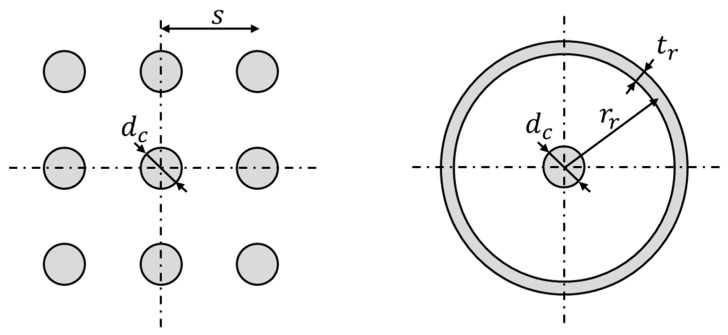
Transformation of stone columns in equivalent gravel rings.

The idea of transforming the stone columns into gravel rings, tubes or cylindrical trenches follows the same concept as for the longitudinal gravel trenches (Section 4) but for problems with axial symmetry rather than plane strain conditions. Thus, the model is used for problems with axial symmetry, such as circular embankments or circular storage tanks. The model implies the transformation of each group of columns into an equivalent gravel ring with the same area to keep $a_{r}$ constant (Figure 11). In the case of Figure 11, the 8 neighboring columns of the central column are transformed into an equivalent ring with a thickness ($t_{r}$) that gives the same area. Assuming that $t_{r}$ is small enough, its value is:

(6) $$t_{r}=d_{c}^{2}/r_{r}$$

The distance of the gravel ring to the central point ($r_{r}$) is the weighted average of the distance of the 8 columns to the center.

(7) $$r_{r}=\left(1+\sqrt{2}\right)s/2$$

For a group of only 4 columns, the weighted average is directly $r_{r}=s$ [58]. The proposal of other authors [59] is slightly different and implies that the gravel ring surrounds the same area as that of the square formed by the 8 columns:

(8) $$r_{r}=2s/\sqrt{\pi }$$

For practical purposes, the differences are negligible.

Mitchel and Huber [58] seem to be the first authors to use this model. They used it in combination with the finite element method to study a field case (the foundation of a water treatment plant). Contrary to the model of the longitudinal gravel trenches, here the confining conditions of the gravel rings seem to be somehow similar to those of the stone columns and it is enough to maintain the values of $a_{r}$ and of the drained properties of the soil and the columns to obtain satisfactory results [60,61]. As for the longitudinal gravel trenches, there is not yet any satisfactory way to convert the cylindrical encasement (geosynthetic) that surrounds the columns for this case.

## 7. Three-Dimensional Slice of Columns

**Figure 12:**
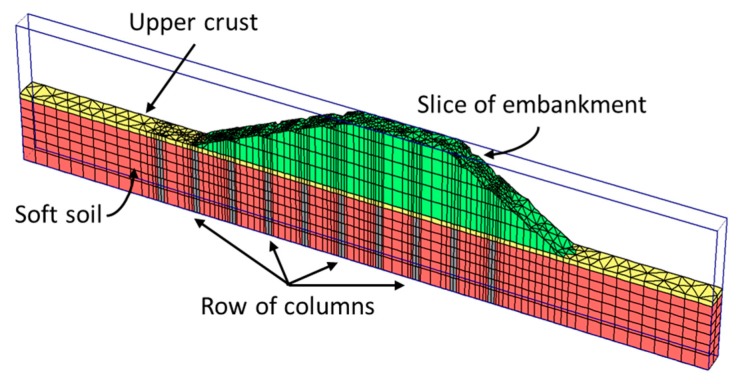
Finite element model of a 3D slice of columns.

There are problems that fulfill plane strain conditions but for the stone column treatment, such as an embankment for a linear infrastructure. In these cases, analyzing a slice or a row of columns is very useful when using 3D numerical analyses (Figure 12). Nowadays, the high computer power and the availability of 3D numerical codes makes this simplified geometrical model very appealing because it is relatively simple and it does not require any transformation or determination of equivalent parameters [48,62]. Furthermore, it allows any of the features or improvements achieved with a stone column treatment to be studied, such as consolidation, deformations and stability (Table 1), and it is valid for both encased and non-encased columns.

**Figure 13:**
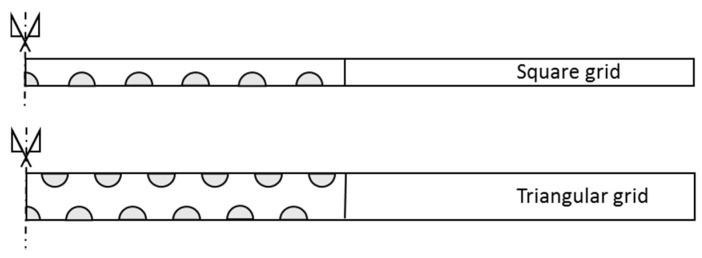
Numerical model of a slice of columns for triangular and square grids.

For the case of a square grid or mesh of columns, it is enough to model just half of a row of columns due to symmetry conditions (Figure 13). On the contrary, for a triangular grid of columns, it is necessary to study at least two different rows of columns (a half of each is enough) (Figure 13).

## 8. Isolated Column

**Figure 14:**
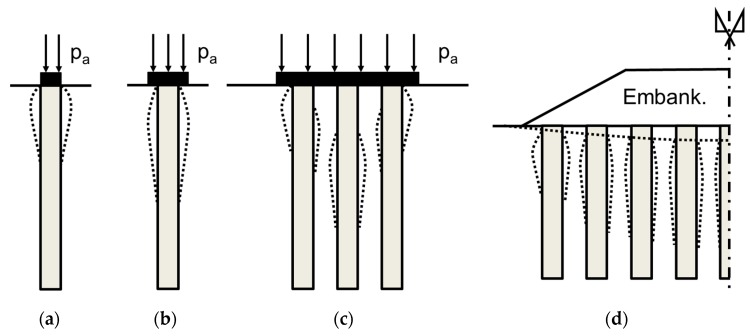
Column deformation for different configurations: (**a**) Isolated column with $a_{r}$ = 100%; (**b**) Isolated column with $a_{r}$ < 100%; (**c**) Groups of columns beneath footing; (**d**) Column treatment beneath an embankment.

For the sake of simplicity, some experimental tests, either in the field or in the laboratory, are performed on an isolated or single stone column (e.g., [63,64]). The load is usually applied only on the column surface (Figure 14a) or on an area slightly higher than the column. In these cases, the area replacement ratio ($a_{r}$) may be defined as the ratio between the area of the column and the loaded area or the footing footprint area [61]. Thus, for isolated columns, $a_{r}$ is usually around 100%, which is not a realistic situation because it is more efficient to increase the loaded area ($a_{r}$ << 100%) [61]. Loading the surroundings of the columns is beneficial because it increases the horizontal stresses at the lateral boundaries of the columns and increases their confinement.

The results of research performed on isolated columns are sometimes directly extrapolated to other cases, leading to some confusion and non-accurate predictions. Some authors (e.g., [15] (p. 28)) show that the column bulges (or notably expands radially) at their upper part, specifically at an upper zone with a length of two or three times the column diameter (2–3 $d_{c}$). Hughes and Withers [63] measured a length of 4 column diameters for the upper bulging zone for an isolated column ($a_{r}$ = 100%) through laboratory tests. That is valid for cases with load only on the single column, i.e., $d_{c}=B$. In the next section, it will be shown that for other cases, it is more meaningful to define the bulging zone using the footing width (B) instead of the column diameter [61] because in those cases, the column bulging may be deeper [3] (p. 114) [15] (p. 28) (Figure 14). Besides, the failure mechanism of the columns may not be bulging, for example, it may be shearing [65].

Isolated columns are generally used to study their bearing capacity. The maximum vertical stress that a stone column may bear is usually given as:

(9) $$\sigma_{vc}^{max}\approx 20c_{u}$$

where $c_{u}$ is the undrained shear strength of the surrounding soft soil. Equation (9) is derived assuming that the column is in an active state with an active earth pressure coefficient of around $k_{ac}$ = 1/3 and that the radial cavity expansion factor is around 6–8 (e.g., [63,66]). Equation (9) is strictly valid for isolated columns with $a_{r}$ = 100%, but when used for larger loaded areas, it is conservative because it neglects the increase in the radial stress due to vertical loading of the soil surrounding the column. Besides, Equation (9) assumes perfect undrained conditions for the soil surrounding the column and some drainage could be expected near the granular column. For common cases, Equation (9) gives a vertical load supported for each stone column of 20–50 tons [15] (p. 6). Sometimes, field tests are performed to check the exact vertical load supported by one isolated column with $a_{r}$ = 100%. The approach of considering the bearing capacity of a footing as the sum of the contribution of each column may usually be overconservative. For footings, an improvement for field tests (plate load tests) on an isolated column may be achieved by using a larger loaded area, so $a_{r}$ is the same as in the footing [3] (p. 165).

## 9. Groups of Columns

**Figure 15:**
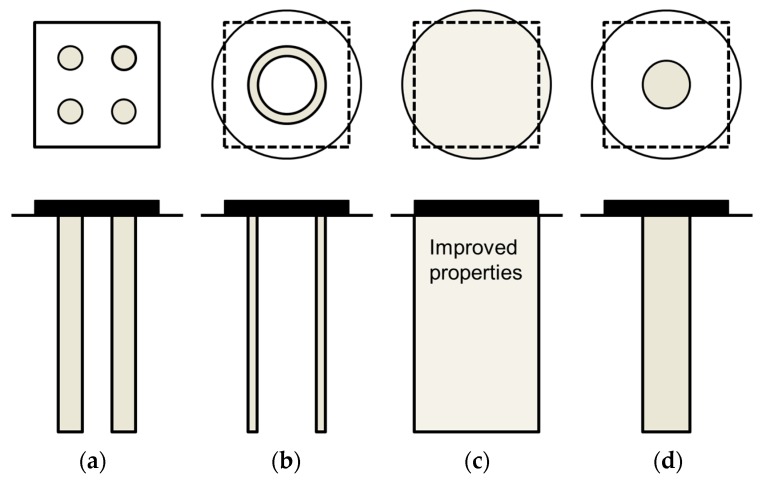
Simplified geometrical model for groups of stone columns beneath a square footing: (**a**) 3D model; (**b**) Gravel rings; (**c**) Homogenization; (**d**) One central column.

Stone column treatments are traditionally used beneath large loaded areas, such as embankments, but they are also used beneath footings, when the applied loads are not high [67]. For these cases, the homogenization technique is valid [68]. Besides, the plane strain model using gravel trenches (Figure 1c) is also valid for strip footings. On the other hand, when the footing could be converted into a circular footing (e.g., a square footing), the simplified geometrical model in axial symmetry that uses gravel rings is also applicable [60,61] (Figure 15). For the latter case (i.e., axial symmetry conditions), the author has recently proposed an additional simplified model that assumes that all the columns may be converted into a central column with the same area (Figure 15d), keeping $a_{r}$ constant [61]. The main advantages of this model are that it may be used to developed analytical solutions [38] and that it is also applicable to encased columns. For encased columns, it is also necessary to transform the elastic properties of the encasement, so that the factor $J_{g}/r_{c}$ is kept constant [69]. The validity of this central column model for small groups of columns is based on the small influence on the footing settlement (usually less than 10%) of the number of columns and their position beneath the footing [61]. This simplified central column is only valid to study the footing settlement and should not be used for other features, such as the consolidation process or the bending moments in the footing.

## 10. Critical Column Length

The concept of critical length is used for stone columns as in piles (e.g., [65]). Thus, for columns longer than the critical length, the settlement reduction or the bearing capacity of the footing does not notably change or improve. For non-encased stone columns, the critical length of the columns for settlement reduction is around 1.5−2 B [61,70]. The critical length for encased columns is usually higher, around 2−3.5 B for common situations [69]. The main controlling parameter of the critical column length is the extension of the load, i.e., B, but the specific value of the critical column length also depends on other parameters, such as $a_{r}$ and the soil and column properties [61,65,69,70].

It is worth noting that many authors (e.g., [71,72,73,74]) give critical lengths as a function of the column diameter, but the column length to diameter ratio has a minor influence by itself [61,69]. The origin of the misinterpretation could lie in the fact that the first proposals [63,64] were for the case of an isolated column with load only on the column surface, i.e., $d_{c}=B$.

**Figure 16:**
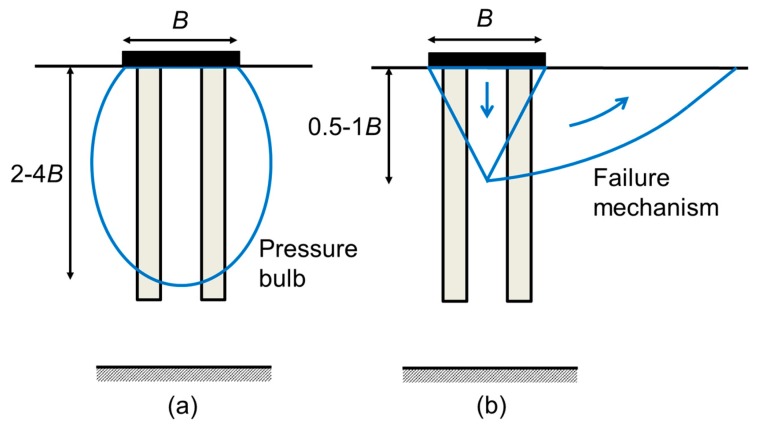
Conceptual justification of critical column length in a homogenous soil layer: (**a**) for settlement reduction; (**b**) for bearing capacity.

For settlement reduction, the critical column length is related to the pressure bulb that the footing generates (Figure 16a), while for footing bearing capacity, the critical length depends on the failure mechanism (Figure 16b). As the critical length for settlement reduction is longer, that is usually the considered value. For large loaded areas (high values of B), the critical column length is higher than the soft layer thickness, and consequently, the concept of critical or optimal length does not apply.

The mechanisms in Figure 16 also explain the influence of additional columns outside the footing. Columns outside the footing are crossed by the slip line or failure mechanism; so, they contribute to the bearing capacity. On the contrary, they hardly influence the settlement reduction if they are outside the pressure bulb. The only minor beneficial effect for the settlement reduction is that they distribute the applied load over a slightly wider area. Therefore, when the bearing capacity is defined in terms of a critical settlement, the effect of extra columns outside the footing is only marginal [65].

The presented analysis on critical column length is based on a homogeneous soft soil layer; for layered soils, this analysis is not directly applicable.

## 11. Column Installation Effects

Column installation alters the surrounding soil, especially when the columns are installed by vibrodisplacement. However, it is commonly accepted that in a stone column treatment, installation effects are less significance and the main improvement is caused by the inclusion of gravel. The search for more accurate designs has led to more detailed analyses of the installation effects [75,76,77,78]. Notable excess pore pressures are consistently measured in the field due to column installation [79]. However, in most cases, these pore pressures quickly dissipate [80]. Column installation, especially by vibrodisplacement, also leads to an increase in the horizontal stresses [75]. This is usually quantified through the earth pressure coefficient, K [3,75,76]. The increase in effective mean stress leads, in turn, to an increase in the soft soil stiffness [3] (p. 138).

The excess pore pressures and the remolding caused by column installation lead to an instantaneous reduction of the undrained shear strength just after installation [77,81]. As the pore pressures dissipates and the effective mean stress increases, the value of the undrained shear strength recovers and usually increases above its initial value [77,81]. Special care must be taken with soil remolding in sensitive soils [67,77]. Poker vibration is necessary for proper column compaction, but excessive use of the poker with many repenetrations (“overworking”) may result in high excess pore pressures and disturbance of sensitive or overconsolidated soil layers, such as a dry, stiff upper crust that is common is soft soils and is beneficial for load distribution [79].

The remolding caused by column installation also alters the permeability of the soft soil in the vicinity of the column. This zone, whose permeability is lower than the natural soft soil, is usually called the “smear” zone [82,83]. The properties of the smear zone, specially its size and permeability, have been largely studied for vertical drains [84,85,86]. Another related problem is column clogging [83]. Gravel compaction during column installation and the high hydraulic gradients at the soil-column interface inevitably produce a migration of clay particles into the pores of the granular column, notably reducing the permeability of an external annulus of the column. This phenomenon is normally less important in geosynthetic encased columns because the geosynthetic usually acts as a filter.

The introduction of installation effects in the numerical modeling of stone columns is a complex process. Some attempts have been made using field measurements and back-fitting the results [75] or using previous numerical analyses that simulate column installation as a cylindrical cavity expansion [78]. From a practical point of view, column installation may be considered altering the at-rest earth pressure coefficient of the natural soft soil. Priebe’s theoretical solution [16] considers, for example, a value of 1. Published values [59] vary between 0.4 and 2.5, with average values slightly above 1. Obviously, the earth pressure coefficient after column installation should depend on the initial value, the construction method and the type of soil. Finally, if the numerical model does not consider a stress-dependent constitutive model for the soft soil, its stiffness should be increased correspondingly [76].

## 12. Properties of the Columns

The modeling process of the soft soil is beyond the scope of this review and it is broadly analyzed elsewhere (e.g., [87,88]). Some authors [79,89] warn about the importance of secondary compression in some soils and the limited capacity of stone columns to reduce it. Gravel properties are also well-analyzed (e.g., [90,91,92]), but the properties of the gravel for stone columns are not so readily available nor are they generally measured for each project. For stone columns, the gravel should be clean, preferably crushed stone, hard, unweathered, free from organics or other deleterious materials and its degradation using the Los Angeles testing machine should be less than a 45% of loss [15] (p. 117). Its grain size should be between 12 and 75 mm. For bottom feed columns, the maximum size is usually limited to 50 mm to avoid obstructions in the feeding tube.

The relative density of the gravel in the stone columns is not usually measured and it may vary along the length of the column, in a similar manner as the column diameter. A proper stone column construction should achieve relative densities of the gravel above 75% [15]. Herle et al. [93] measured values close to 100%.

The friction angle of the columns ($\phi_{c}$) has a notable influence on the results of a stone column treatment. Its value decreases with the confining pressure ($\sigma_{c}$). Using samples in a dense state, Herle et al. [93] measure values up to 60° for low confining pressures ($\sigma_{c}$ = 50 kPa) and values around 50° for medium confining pressures ($\sigma_{c}$ = 200 kPa) (Table 2). However, when choosing a value of the friction angle to model stone columns, it is necessary to consider the following points:

- Columns are usually under triaxial conditions and for granular soils, the friction angle measured by direct shear tests is slightly higher than that measured using triaxial tests [94].
- Values in Table 2 are peak values, but plastic strains in the columns may be important; so, it is recommended to reduce peak friction values by 5–7% to obtain approximate residual values [3].
- The validity of some theoretical methods and their success in predicting field measurements may be linked to the use of conservative values of the friction angle.
- Published numerical analyses consider values of 40–45° (Table 3).

The Young’s modulus of the columns is usually between 25 and 100 MPa (Table 3) and it also varies with the confining pressure. A hyperbolic power law is sometimes used to reproduce the stress dependent stiffness of the gravel of the columns (e.g., [58,95,96]). The common value of the exponent of the power law is around 0.3 (Table 3). The unit weight of the gravel does not require further comment, except for the cases where the columns do not reach a rigid substratum and they are installed by vibrodisplacement (e.g., [49]). In those cases, the addition of gravel and its corresponding weight may cause additional deformation in the underlying layer that is not improved with stone columns. For example, [49] used non-realistic high values, namely $\gamma_{sat}$ = 23.5 kN/m^3^ to account for this effect.

Regarding encased stone columns, the material used to construct them is usually sand instead of gravel. The available information is more limited, but it seems obvious to use lower values of both the friction and dilatancy angles (Table 4).

## 13. Modeling the Geosynthetic Encasement

As shown in Figure 4, the geosynthetic encasement may be modeled as a flexible membrane that does not support compressive stresses, has a negligible thickness and behaves as a linear elastic material with a modulus of $J_{g}$ = 1000–5000 kN/m. Regarding its tensile strength, for modeling purposes, it may usually be enough to verify that it is far from being reached. The tensile strength is usually reached for circumferential strains of around 5–10%, which implies strength values of 100–300 kN/m [5]. It is common that geosynthetic may be anisotropic and then different properties should be input for each direction.

Some other geosynthetic features, such as creep and damage during installation, are usually considered through reduction factors. Recent numerical analyses [99] model numerically the creep behavior of geosynthetics.

In those numerical analyses where the geosynthetic is modeled as a continuum element of small thickness, it is necessary to ensure that it does not support compressive stresses, and hence, bending moments. Its Young’s modulus should be $J_{g}$ divided by its thickness.

Little attention is usually paid to the Poisson’s ratio of the geosynthetic ($\nu_{g}$), but it may have important consequences on the results [100]. When the encasement is modeled as a membrane, it is common to study the two directions of the geosynthetic independently, without interaction between them (Equation (3)). That means $\nu_{g}$ = 0.

On the other hand, when the geosynthetic is modeled as a continuum element, it is necessary to specify a value for $\nu_{g}$. In some cases (e.g., [31]), a common value of 0.3 is input. That implies that, although the geosynthetic that surrounds the columns does not support compressive stresses in the vertical direction, it compresses vertically (vertical strains) and then, that vertical compression leads to a radial expansion of the geosynthetic (horizontal or circumferential strains) due to Poisson’s effect. Thus, the geosynthetic encasement decreases its lateral confinement to the column due to this radial expansion. Common geosynthetics for column encasement are woven geotextiles. For woven geotextiles, the two directions work nearly independently; so, it seems logical to use values of the Poisson’s ratio close to 0. Soderman and Giroud [101] propose values of $\nu_{g}$ = 0.1 for woven geotextiles and $\nu_{g}$ = 0.35 for non-woven geotextiles.

## 14. Conclusions

This paper reviews the main modeling techniques for stone columns, both ordinary or non-encased stone columns, and geosynthetic-encased stone columns. The paper tries to encompass the more recent advances and recommendations in the topic.

There exist several simplified geometrical models. The suitability of each of them depends on the type of process to be studied, e.g., bearing capacity or settlement, and the type of analyses, e.g., analytical or numerical in 2D or 3D. For numerical analyses of a problem that fulfils plane strain conditions but for the stone column treatment, such as an embankment for a linear infrastructure, the simplified geometrical model of a three-dimensional slice of columns is recommended because it does not require any transformation of the problem parameters. For more simplified models for the stone column treatment, such as gravel trenches or homogenization, calibrating or tuning the parameters using the unit cell as an auxiliary problem is recommended. The model of an isolated column with load just on top of it may be useful for field tests, but it is not a common situation in real cases because loading the soil that surrounds the column is beneficial. The behavior of an isolated column is different from that of a column within a large group under distributed load. Those differences should be considered when extrapolating results.

For groups of columns, the critical column length depends mainly on the loading area. For non-encased columns, its value is around twice the footing width and for encased columns is slightly higher.

Some guidance is provided to consider installation effects and to model the column and the geosynthetic encasement. Column installation usually increases the horizontal stresses and that is often accounted for using a high value of the earth pressure coefficient, typically around 1. On the other hand, the relevance of the Poisson’s ratio of the geosynthetic is taken into account and an appropriate value for a woven geotextile could be close to 0, i.e., the two directions work nearly independently.

Finally, laboratory tests are not commonly performed to characterize stone column properties and some column parameters published in the literature are tabulated as a reference.

## Figures and Tables

**Table 1:** Suitability of simplified geometrical models to study different features of a stone column treatment for the foundation of an embankment.

Geometrical Model	Final Settlement	Consolidation	Stability
Unit cell	***	***	-
Gravel trenches	**	**	**
Homogenization	**	*	*
3D slice	***	***	***

*** Completely suitable, ** Moderately suitable, * Slightly suitable, - Not suitable.

**Table 2:** Stress-dependent peak friction angles of dense gravel [93].

Type of Gravel	$\phi_{c,max}$ (°)	$\sigma_{c,max}$ (kPa)	$\phi_{c,min}$ (°)	$\sigma_{c,min}$ (kPa)	Remarks
Crushed lime stone	63.1	50	53.8	200	DS, Vibro SC
River gravel	58.8	50	51.9	200	DS, Vibro SC
River gravel, sub-round	57.1	50	50.9	200	DS, Vibro SC, $d_{60}/d_{10}$ = 2.6
Rivel gravel, sub-round	59.2	50	53.2	200	DS, Vibro SC, $d_{60}/d_{10}$ = 2.1
Rivel gravel, crushed	60.4	50	55.2	200	DS, Vibro SC
Dolomite	64.0	15	43	500	TX, γ = 17 kN/m^3^, [90]
Dolomite	54.0	15	40	500	TX, γ = 15 kN/m^3^, [90]
Sandstone	60.1	27	45.6	695	TX, [92]
Basalt	64.2	27	45.6	695	TX, [92]
Basalt	71.8	8	45.6	240	TX, $d_{50}$ = 30 mm, [91]
Basalt	70.0	8	51.1	120	TX, $d_{50}$ = 30 mm, [91]

DS: Direct shear test; TX: Triaxial test; Vibro SC: dense gravel for vibrated stone columns [93]; $d_{60}/d_{10}$: uniformity coefficient.

**Table 3:** Parameters used to model stone columns in numerical analyses.

Reference	$\phi_{c}$ (°)	$\psi_{c}$ (°)	$E_{c}$ (MPa)	ν (-)	m (-)	$\gamma_{d}$/$\gamma_{sat}$ (kN/m^3^)
[59]	41	-	29.2	0.2	0.59	18.6/21.6
[68]	35	-	67.5	0.3	-	-
[70]	40–35	3	50	0.3	-	-
[74]	45	0	100	0.3	-	19/19
[73]	48	26	2.5	0.3	-	16/-
[30]	40	-	30	0.3	-	15/15
[54]	40	0	30	0.3	-	-/15
[49]	35	5	25	0.2	0.3	20/23.5
[78]	42	12	35	0.2	-	16/19
[95]	46	10	22	0.15	0.25	-
[96]	45	15	70	0.2	0.3	19/19

The reference pressure for the stiffness is 100 kPa; m: Exponent of the power law used to reproduce the stress dependent stiffness.

**Table 4:** Parameters used to model encased stone columns in numerical analyses.

Reference	$\phi_{c}$ (°)	$\psi_{c}$ (°)	$E_{c}$ (MPa)	ν (-)	m (-)	$\gamma_{d}$/$\gamma_{sat}$ (kN/m^3^)
[73]	48	4	9	0.3	-	16/-
[97]	40	10	40	0.3	-	-/23
[98]	38	5	15.5	0.3	-	-/18
[28]	40	-	80	-	-	-/20
[5]	32.5	0	-	-	-	19/20
[31]	35	0	30	0.2	-	-
[48]	38	10	40	0.3	-	-/22
[69]	40	5	30	0.33	-	-/20

m: Exponent of the power law used to reproduce the stress dependent stiffness.

## List of Symbols

*a_r_*	Area replacement ratio: $A_{c}/A_{e}$
*c_u_*	Undrained shear strength
*d_c_*	Column diameter
*d*_10_, *d*_60_	Diameters corresponding to 10% and 60% finer in the particle-size distribution, respectively
*k*	Hydraulic conductivity/permeability
*m*	Exponent of the power law used to reproduce the stress dependent stiffness
*p_a_*	Uniform applied vertical pressure
*p_a_*(0)	Uniform applied vertical pressure at the surface
*p_a_*(*z*)	Reduced value using a trapezoidal distribution of the uniform applied vertical pressure at a depth z
*r*	Radius
*s*	Centre-to-centre column spacing
*s_r_*	Radial displacement of the column
*s_z_*	Settlement
*s*_*z*0_	Settlement without columns
*t*	Thickness
*u*	Pore water pressure
*A*	Cross-sectional area
*B*	Footing width
*E*	Young’s modulus
*H*	Soft soil layer thickness
*J_g_*	Encasement stiffness
*K*	Coefficient of lateral earth pressure
*K*_0_	Coefficient of lateral earth pressure at rest
*L*	(Column) length
*T_g_*	Tensile hoop force at the encasement
*b*	Settlement reduction factor: $\beta =s_{z}/s_{z0}$
*g*	Unit weight
*ν*	Poisson’s ratio
*σ*	Stress
*σ_c_*	Confining pressure
*φ*	Friction angle
*ψ*	Dilatancy angle

## Subscripts

*c*, *s*, *g*, *e*	column, soil, encasement or geosynthetic, loaded area or unit cell
*d*	dry
*m*	equivalent homogeneous soil
*max*	maximum value
*min*	minimum value
*r*	equivalent gravel ring
*r*, *θ*, *z*	Cylindrical coordinates
*sat*	Saturated
*x*, *y*, *z*	Cartesian coordinates
^¯^ (upper bar)	average value along the radius

## Abbreviations

DS	Direct shear test
G.W.T.	Ground water table
SC	Stone column
TX	Triaxial test
